# The glutaminase (*Cg*GLS-1) mediates anti-bacterial immunity by prompting cytokine synthesis and hemocyte apoptosis in Pacific oyster *Crassostrea gigas*

**DOI:** 10.1038/s41598-020-80552-2

**Published:** 2021-01-14

**Authors:** Yage Liang, Meijia Li, Zhaoqun Liu, Yuanmei Li, Lingling Wang, Linsheng Song

**Affiliations:** 1 Liaoning Key Laboratory of Marine Animal Immunology, Dalian Ocean University, Dalian, 116023 China; 2 Southern Marine Science and Engineering Guangdong Laboratory, Zhuhai, 519000 China; 3 Functional Laboratory of Marine Fisheries Science and Food Production Processes, Qingdao National Laboratory for Marine Science and Technology, Qingdao, 266235 China; 4 Liaoning Key Laboratory of Marine Animal Immunology and Disease Control, Dalian Ocean University, Dalian, 116023 China; 5 Dalian Key Laboratory of Aquatic Animal Disease Prevention and Control, Dalian Ocean University, Dalian, 116023 China

**Keywords:** Innate immunity, Marine biology

## Abstract

Glutaminase, an amidohydrolase enzyme that hydrolyzes glutamine to glutamate, plays crucial roles in various immunomodulatory processes such as cell apoptosis, proliferation, migration, and secretion of cytokines. In the present study, a glutaminase homologue (designated as *Cg*GLS-1) was identified from Pacific oyster *Crassostrea gigas*, whose open reading frame was of 1836 bp. *Cg*GLS-1 exhibited high sequence identity with vertebrate kidney-type GLS, and closely clustered with their homologues from mollusc *C. virginica*. The enzyme activity of recombinant *Cg*GLS-1 protein (rCgGLS-1) was estimated to be 1.705 U/mg. *Cg*GLS-1 mRNA was constitutively expressed in all the tested tissues of oysters, with the highest expression level in hemocytes. *Cg*GLS-1 mRNA expression in hemocytes was significantly up-regulated and peaked at 6 h (2.07-fold, *p* < 0.01) after lipopolysaccharide (LPS) stimulation. The *Cg*GLS-1 protein was mainly distributed in the cytoplasm with a significant co-location with mitochondria in oyster hemocytes. The content of Glu in the oyster serum was significantly decreased after the inhibition of *Cg*GLS-1 using specific inhibitor Bis-2- [5-(phenyl acetamido)-1,3,4-thiadiazol-2-yl] ethyl sulfide (BPTES), and the expression levels of *Cg*mGluR6, *Cg*AP-1, cytokines *Cg*IL17-5 and *Cg*TNF-1 were significantly decreased after BPTES and LPS stimulation. The transcripts of *Cg*Caspase3 as well as the apoptosis index of hemocytes were also decreased. These results collectively suggest that *Cg*GLS-1 is the enzyme to synthesize Glu in oyster, which can modulate anti-bacterial immunity by regulating the secretion of pro-inflammatory cytokines *Cg*IL17-5 and *Cg*TNF-1, as well as hemocyte apoptosis.

## Introduction

Phosphate-activated glutaminase hereinafter, referred to as GLS, is an amidohydrolase enzyme catalyzes the reaction of glutamine (GLN) to glutamate (Glu) and ammonia^1^. Two genes in chromosome 2 and 12 of humans encode tissue-specific isoenzymes of glutaminase. One located in chromosome 2 encodes the kidney-type isozyme, and another located on chromosome 12 encodes the liver-type isozyme^2^. Liver-type GLS is expressed only in periportal hepatocytes of the postnatal liver, where it takes part in hepatic ureagenesis^3^. Kidney-type GLS is widely found in tissues such as kidney, brain and even lymphocytes, where the resulting ammonia is directly released without urea generation^3^. Their structural and kinetic characteristics are also different from each other, which contribute to their function and short-term regulation^3^. As a multifaceted protein, GLS plays a crucial role in some physiological processes in mammals, not only ammonia and urea genesis, but also synthesis of neurotransmitter Glu^4–10^. Glu synthesized by GLS functions not only as neurotransmitter, but also as an important immunomodulator^11^.

Glu can bind directly to its receptors on immune cells and induce various immune reactions. As the important information molecule between the immune system and the nervous system, Glu plays a crucial role in the initiation and development of adaptive immune responses in vertebrates^12^. For example, the Glu at low level could bind ionotropic Glu receptor3 (GluR3) to increase T cell adhesion and chemotactic migration^13^, while the excess Glu could activate metabotropic Glu receptor5 (mGluR5) to decrease the proliferation of T cells and activate mGluR1 to induce cytokine secretion^13,14^. It is reported that Glu contributes to the improved/prolonged T cells survival by protecting them from apoptosis^15^. The locomotion and apoptosis of cell was inhibited after the blockage of α-amino-3-hydroxy-5-methyl-4-isoxazole-propionicacid ionotropic receptor (AMPA iGluR), a Ca^2+^-permeable receptor^16^. Glu can modulate immune response by regulating the secretion of several cytokines. The high contents of Glu (10^–3^ M) is able to induce interferon (IFN)γ and interleukin (IL)-10 secretion in the T cells activated by anti-CD3^17^. In contrast, Glu at ~ 1,000-fold lower content of 1 × 10^–6^ M could regulate mGluRs to modulate IL-6 production and enhance the secretion of tumor necrosis factor (TNF)-α, IFNγ, IL-2, and IL-10^18^. These findings indicate that Glu is involved in regulating the function and survive of the immune cells, and ultimately contribute to the protection of the host from invading pathogens.

As the most abundant excitatory neurotransmitter in the brain of vertebrates, Glu together with its receptors and GLS, are also identified in invertebrates such as insects, round worm, and platyhelminths^19^. For example, Glu has been detected in many invertebrates such as *Drosophila*^20^, sponge^21^, and ctenophore^22^. Glutamate receptors have been cloned from nematodes^23,24^ and insects^25^. Glu also plays important roles in neural and immune responses of invertebrates. In the phylogenetically basal hydrozoan *Hydra vulgaris*, Glu induced the outputs of ectodermal and endodermal impulse generating systems^26^, and acted as an excitatory neurotransmitter in cestode and aplysiidae nervous systems^27^. As the main excitatory neurotransmitter in the nervous system, Glu also cooperates with inhibitory neurotransmitter GABA to maintain the homeostasis of immune response in invertebrates. It was found in previous study that the immune response level of *C. gigas* could be adjusted by regulating the balance between Glu and GABA^28^. However, the information about the modulation of glutaminergic system on the response of immune cells in molluscs is still far from well understood.

Pacific oyster *C. gigas* is an important cultured mollusc species, which contributes weightily to the aquaculture industry worldwide. They live in the coastal and estuarine areas harboring a large diversity of bacteria, which might be one of the important inducers of the diseases^29^. Evaluating the response mechanism to the invading bacteria would be helpful for the development of disease control strategies for the oyster aquaculture. It has been reported that amino acid neurotransmitters play an important role in regulating the immune response of molluscs^28,30^. In the present study, a homologue of GLS was identified from *C. gigas* (designated as *Cg*GLS-1) with the main purposes to (1) examine its mRNA expression level in different tissues, and in the response against bacterial stimulation, (2) determine its enzyme activity to catalyze the hydrolytic deamidation of glutamine to Glu, (3) examine the apoptotic rate, Glu concentration and mRNA expression of *Cg*IL17-5, *Cg*TNF-1 and *Cg*Caspase3 in oyster hemocytes after the inhibition of *Cg*GLS-1, hope to explore the function of GLS in bacteria-induced immune response in oyster.

## Materials and methods

### Oysters, treatments and sample collection

The Pacific oysters *C. gigas* (about 2-year old, averaging 150 mm in shell length) were collected from a local farm in Dalian, Liaoning Province, China, and cultured in aerated seawater at approximately 22 °C for a week of acclimation before processing.

Nine oysters without any treatment were employed to investigate the distribution of mRNA in tissues including hepatopancreas, adductor muscle, gonad, gill, visceral ganglia, mantle, as well as hemocytes. The same samples from three oysters were pooled together as one replicate, and there were three replicates for each tissue.

One hundred and eight oysters were randomly divided into seawater (SW) group and LPS group. Fifty-four oysters in SW group received an injection of 100 μL of sterilized seawater according to previous description^31^. Another fifty-four oysters in LPS group received individual injection of 100 μL lipopolysaccharide (0.5 g/L in sterilized seawater, from *Escherichia coli* O111:B4, Sigma Aldrich, USA). Nine oysters were randomly sampled from each group at 0, 6, 12, 24, 48 and 72 h after stimulation. The hemocytes were collected according to previous description^28^, and the same samples from three individuals were pooled together as one replicate. There were three replicates for each time point. In brief, the hemolymph was centrifuged at 800 × *g*, 4 °C for 15 min to harvest hemocytes and serum. The hemocytes were harvested for RNA extraction, cDNA synthesis and quantitative real-time PCR (qRT-PCR).

Another 108 oysters were employed for the enzyme inhibitor incubation assay, and they were divided into three groups including SW, dimethyl sulfoxide (DMSO) and Bis-2- [5-(phenyl acetamido)-1,3,4-thiadiazol-2-yl] ethyl sulfide (BPTES) group. BPTES is a selective kidney-type glutaminase (glutaminase 1, GLS1, KGA) inhibitor (Sigma-Aldrich)^32,33^. It was dissolved in DMSO at a final concentration of 1.5 g/L and stored at -20 °C as stock solution as previous description^34^. The oysters in each group received an injection of 100 μL of sterilized seawater, 100 μL DMSO and 100 μL BPTES (1.5 g/L), respectively. The serum was collected from nine oysters in each group at 0, 1, 6 and 12 h post injection following the description above. There were three replicates for each time point. The serum was used for the determination of Glu concentration.

For the enzyme inhibitor incubation and LPS stimulation experiment, 135 oysters were divided equally into three groups including DMSO + SW, DMSO + LPS and BPTES + LPS group. They were firstly treated as previous description with 100 μL DMSO, 100 μL DMSO and 100 μL BPTES (1.5 g/L), and then received individually a second injection of 100 μL sterilized sea water, 100 μL LPS (0.5 g/L) and 100 μL LPS (0.5 g/L), respectively, at 6 h after the first injection. Hemocytes from nine oysters in each group were collected at 0, 6, 12, 24 and 48 h post the second injection, and three of them were pooled together as one replicate. The hemocytes were used for apoptosis assay and the total RNA extraction for RT-PCR analysis of *Cg*IL17-5, *Cg*TNF-1, *Cg*AP-1, mGluR6, and *Cg*Caspase3 expression. The serum was used for the determination of Glu concentration as above description.

### RNA isolation and cDNA synthesis

Total RNA was isolated from oyster tissues using TRIzol reagent according to the standard protocol (Invitrogen)^35^. RNA concentration was measured by a NanoDrop reader (Saveen & Werner ApS, Denmark)^36^, and the integrity and purity of RNA were examined by electrophoresis analysis in 1.0% agarose gel. The total RNA was then treated with DNaseI (Promega) to remove trace DNA contamination. The synthesis of the first-strand cDNA was carried out with Promega M-MLV RT with oligo (dT)-adaptor priming according to the manufactory’s protocol^35,36^. The synthesis reaction was performed at 42 °C for 1 h, terminated by heating at 95 °C for 5 min^35,36^. The cDNA mix was diluted to 1:50 and stored at − 80 °C for subsequent SYBR Green fluorescent quantitative real-time PCR.

### Gene cloning and sequence analysis of *Cg*GLS-1

Blastp analysis of all oyster protein sequences in NCBI database revealed that one sequence (CGI_10008856, named as *Cg*GLS-1) was homologous to glutaminase identified previously in vertebrates. The full-length cDNA of *Cg*GLS-1 was cloned from a cDNA library using specific primers (Table 1). Homology searches of the cDNA sequence and protein sequence of *Cg*GLS-1 were conducted with BLAST algorithm at the National Center for Biotechnology Information (http://www.ncbi.nlm.gov/blast)^36,37^. The deduced amino acid sequence was analyzed with the Expert Protein Analysis System (http://www.expasy.org)^36^. The protein domain was predicted with the simple modular architecture research tool (SMART) version 5.1 (http://www.smart.emblheidelberg.de/)^36,37^. Multiple sequence alignment of the *Cg*GLS-1 with other GLSs was created by the ClustalW multiple alignment program (http://www.ebi.ac.uk/Tools/clustalw2/) and multiple sequence alignment show program (http://www.biosoft.net/sms/index.html)^36,37^. The Neighbor-Joining (NJ) phylogenetic tree was constructed using the MEGA 6.0 package with 1,000 pseudo-replicates of bootstrap resampling to test the reliability of the branching.

**Table 1:** Sequences of the primers used in this study.

Primer name	Sequence (5′-3′)
**Clone primers**	
P1 Oligo (dT)-adaptor	GGCCACGCGTCGACTAGTACT
P2 *Cg*GLS-1-F	Forward: ATGTATAAATACCTTCGTGACTT
P3 *Cg*GLS-1-R	Reverse: TTAATCCGTCTCTGGATGCT
**Recombinant expression**	
P4 M13-47	Forward: CGCCAGGGTTTTCCCAGTCACGAC
P5 RV-M	Reverse: GAGCGGATAACAATTTCACACAGG
P6 *Cg*GLS-1-30a-F-BamHI	Forward: CGGGATCCATGTATAAATACCTTCGTGACTT
P7 *Cg*GLS-1-30a-R-HindIII	Reverse: CCCAAGCTTATCCGTCTCTGGATGCT
**RT-PCR primers**	
P8 *Cg*GLS-1-RT-F	Forward: TTTATCAGAACGAGAAACGGC
P9 *Cg*GLS-1-RT-R	Reverse: CCATTAGCGAGGGTAGCAGCA
P10 EF-RTF	Forward: AGTCACCAAGGCTGCACAGAAAG
P11 EF-RTR	Reverse: TCCGACGTATTTCTTTGCGATGT
P12 *Cg*IL17-5-F	Forward: CGTCCTTGCCTTACTGACTAGA
P13 *Cg*IL17-5-R	Reverse: TGTCGTTGTCCTCTACCATGAT
P14 *Cg*TNF-1-F	Forward: CTTCTCGTCTGCGGCTTCTTT
P15 *Cg*TNF-1-R	Reverse: CAGGGCTGCGGTCTTTCC
P16 *Cg*Capase3-F	Forward: CGGGAAATTACGGGGAGTTG
P17 *Cg*Capase3-R	Reverse: TCTTCGGAGGATACAGAGGG
P18 *Cg*AP-1-RT-F	Forward: CTTCAGGTCCCCAGTCATTA
P19 *Cg*AP-1-RT-R	Reverse: GGGTAGGATTCCGTCAGTG
P20 *Cg*mGluR6-RT-F	Forward: TTCGTTTTGTGAAAGGCAGGG
P21 *Cg*mGluR6-RT-R	Reverse: GGCTTACAGTCCCAGCAACAG

### Real-time PCR analysis of *Cg*GLS-1, *Cg*IL17-5, *Cg*TNF-1 and *Cg*Caspase3

The mRNA expressions of *Cg*GLS-1, *Cg*IL17-5 (GenBank accession No. KJ531896)^39^, *Cg*TNF-1 (CGI_10005109)^40^, *Cg*AP-1 (CGI_10006579)^41^, mGluR6 (CGI_10011788) and *Cg*Caspase3 (GenBank accession No. EKC34324)^42^ were determined by SYBR Green quantitative real-time PCR method on an ABI PRISM 7500 Sequence Detection System with a total volume of 25.0 μL, containing 12.5 μL of SYBR Green Mix (Takara), 0.5 μL of each primer (10 μmol/L), 2.0 μL of the 50 times diluted cDNA, and 9.5 μL of DEPC-water. The fragment of oyster elongation factor (EF, CGI_10012474) was used as internal control (Table 1). Dissociation curve analysis of amplification products was performed to confirm that only one PCR product was amplified and detected. The comparative average cycle threshold method was used to analyze the expression level of six genes according to the previous report^43^. All data were given in terms of relative mRNA expression using the 2^−ΔΔCt^ method^44,45^.

### Prokaryotic expression and purification of recombinant protein

The cDNA sequence of *Cg*GLS-1 containing glutaminase domain was cloned into pET-30a vector (Primers were shown in Table 1). Restriction enzymes *BamH* I and *Hind* III were used to construct recombinant plasmids. The recombinant plasmid was isolated by MiniBEST plasmid purification kit (Takara, Japan) and then transferred into *E. coli* Transetta (DE3) (Transgen, China). Isopropyl β-D-Thiogalactoside (IPTG) (1 mmol/L) was used to induce the expression of recombinant protein, and the recombinant protein *Cg*GLS-1 (designated r*Cg*GLS-1) was purified by a Ni^2+^chelating Separate column (Sangon Biotech, China). The purity of obtained r*Cg*GLS-1 was evaluated by SDS–polyacrylamide gel electrophoresis. An enhanced BCA protein assay kit (Beyotime, China) was used to quantify the content of r*Cg*GLS-1^46^. The purified protein was stored at −80 °C before use.

### Preparation of polyclonal antibody and Western blot analysis

r*Cg*GLS-1 was renatured by gradient urea-TBS glycerol buffer (50 mmol/L NaCl, 50 mmol/L Tris–HCl, 10% glycerol, 0.2 mmol/L oxide glutathione, 2 mmol/L reduced glutathione, a gradient urea concentration of 6, 5, 4, 3, 2, 1, and 0 mol/L, pH 7.5) at 4 °C for 12 h and dialyzed continuously against ddH_2_O 4 °C for 12 h before it was freeze concentrated. The renatured r*Cg*GLS-1 was immunized to six weeks old rats to acquire polyclonal antibody according to the previous report^47^.

The specificity of polyclonal antibody was identified by Western blot assay. The r*Cg*GLS-1 was separated by 12% SDS-PAGE and then transferred to nitrocellulose membrane (Millipore, USA). The nitrocellulose membrane was soaked in blocking buffer (5% skimmed milk) at 4 °C for 12 h. The nitrocellulose membrane was then incubated with polyclonal antibodies against r*Cg*GLS-1 (diluted 1:700 in 5% skimmed milk) at 4 °C overnight followed by extensive washing, and further incubated with goat anti-mouse IgG conjugated with HRP at room temperature for 1 h. After washed by TBST, the membranes were incubated with Western Lightning-ECL reagent (PerkinElmer, USA), and then exposed to film (Kodak, USA) to visualize the blotted protein.

### Subcellular localization analysis of *Cg*GLS-1 by immunofluorescence assay

Immunocytochemistry of hemocytes was performed according to previous description with some modification^48^. Hemolymphs were collected from three oysters with 10-mL syringe (1.6 × 30-gauge needle) containing equal volume of pre-chilled anticoagulant (6.06 g/L Tris–HCl; 2% glucose, 2% NaCl; 5.84 g/L EDTA; pH 7.4) and immediately centrifuged at 800 × *g*, 4 °C for 15 min to harvest the hemocytes. The hemocytes were resuspended in modified L-15 cell culture media (with additional saline 20.2 g/L NaCl, 0.54 g/L KCl, 0.6 g/L CaCl_2_, 1.0 g/L MgSO_4_, and 3.9 g/L MgCl_2_)^49^, and incubated with Mito-Tracker Red CMXRos (Beyotime Biotechnology, China, C1049) operating fluid (diluted 1:10,000 in L-15 cell culture media) at room temperature for 30 min to stain mitochondria. After centrifuging at 1,000 × *g* for 5 min, the hemocytes were resuspended in L-15 cell culture media again and dropped on polysine microscope adhesion slides (Solarbio, China) for 1 h to form monolayer cells. Then, 4% paraformaldehyde was used to fix the cells. After three times of washing with 0.01 M PBS-T, hemocytes were permeabilized with 0.5% Triton-100 for 5 min, blocked with 3% BSA (Fetal bovine serum albumin diluted in PBS-T) at room temperature for 30 min, and then incubated with the antiserum of *Cg*GLS-1 (diluted 1:1,000 in 3% BSA) at room temperature for 1 h. After washing with TBS-T for three times, hemocytes were incubated with Alexa Fluor 488-conjugated goat anti-mouse secondary antibody (ABclonal, diluted 1:1,000 in 3% BSA) for 1 h. Finally, hemocytes were washed three times with TBS-T before incubation with DAPI (Beyotime Biotechnology, diluted 1:10,000 in PBS) for 5 min, and observed under a laser confocal scanning microscopy (Carl Zeiss LSM 710, Germany).

### Determination of r*Cg*GLS-1 activity

The r*Cg*GLS-1 activity was determined by GLS kit (Jiancheng, China, A124) according to the operation instruction. As r*Cg*GLS-1 can catalyze the hydrolysis of glutamine into L-glutamate and ammonia, the increase rate of ammonia was determined by Nessler's Reagent Spectrophotometry to calculate its enzyme activity. The reaction with the equal volume of final dialysate was employed as negative control reaction. While the reaction with the equal volume of oyster serum was set as positive control reaction. The enzymatic activity of r*Cg*GLS-1 was defined as the amount of ammonia (mol) produced by 1 mg r*Cg*GLS-1 in 1 min (U/mg).

### Glu content measurement

The content of Glu in oyster serum was measured according to the protocol of Glutamate ELISA Kit (Mlbio Shanghai Enzyme-linked Biotechnology, China) based on double antibody one-step sandwich ELISA method. Briefly, 50 μL of serum and 50 μL of HRP (horse radish peroxidase)-labeled antibody of Glu were added into a 96 micro-well plate which was coated with purified anti-Glu antibody. The plate was covered with the closure plate membrane, and the mixture was incubated at 37 °C for 1 h. After washed with washing buffer for three times, 50 μL of Substrate A and 50 μL of Substrate B were added to each well. After incubation in dark at 37 °C for 10 min, 50 μL of Stop Solution was added to each well to stop the reaction. The fluorescence intensity of the mixture was measured at 450 nm (Biotek, USA) within 15 min. The content of Glu in oyster serum was calculated from standard curves generated according to the protocol.

### Determination of hemocyte apoptosis rate by flow cytometry (FCM)

The apoptosis rate of oyster hemocytes was determined by FCM with the Annexin V-FITC/PI double labeling method according to the manual of Annexin VFITC Apoptosis Detection Kit (Beyotime biotechnology, China)^50,51^. The hemocytes were centrifuged at 800 × *g*, 4 °C for 10 min and washed with fresh modified L-15 medium. According to the manufacturer's instructions, 195 μL of the diluted hemocyte (at a final concentration of 5 × 10^5^–10^6^ cells/mL) were incubated with 5 μL of Annexin V-FITC in dark for 10 min to label early-apoptotic cells and then stained with 10 μL of propidium iodide (PI) for 5 min to mark the late-apoptotic or necrotic cells. The hemocyte resuspension was transferred into a polystyrene round-bottom tube and the apoptosis rate was determined by flow cytometry (BD FACS Aria II SORP).

### Statistical analysis

All the data were given as means ± S.D., and analyzed by Statistical Package for Social Sciences (SPSS) 20. Significant differences between treatments for each assay were tested by one-way analysis of variance (ANOVA) and followed by the Duncan’s test. The differences were considered statistically significant at *p* < 0.05, labeled with “*” and extremely significant at *p* < 0.01, labeled with “**”.

## Results

### The sequence characteristics and phylogenetic relationship of *Cg*GLS-1

**Figure 1:**
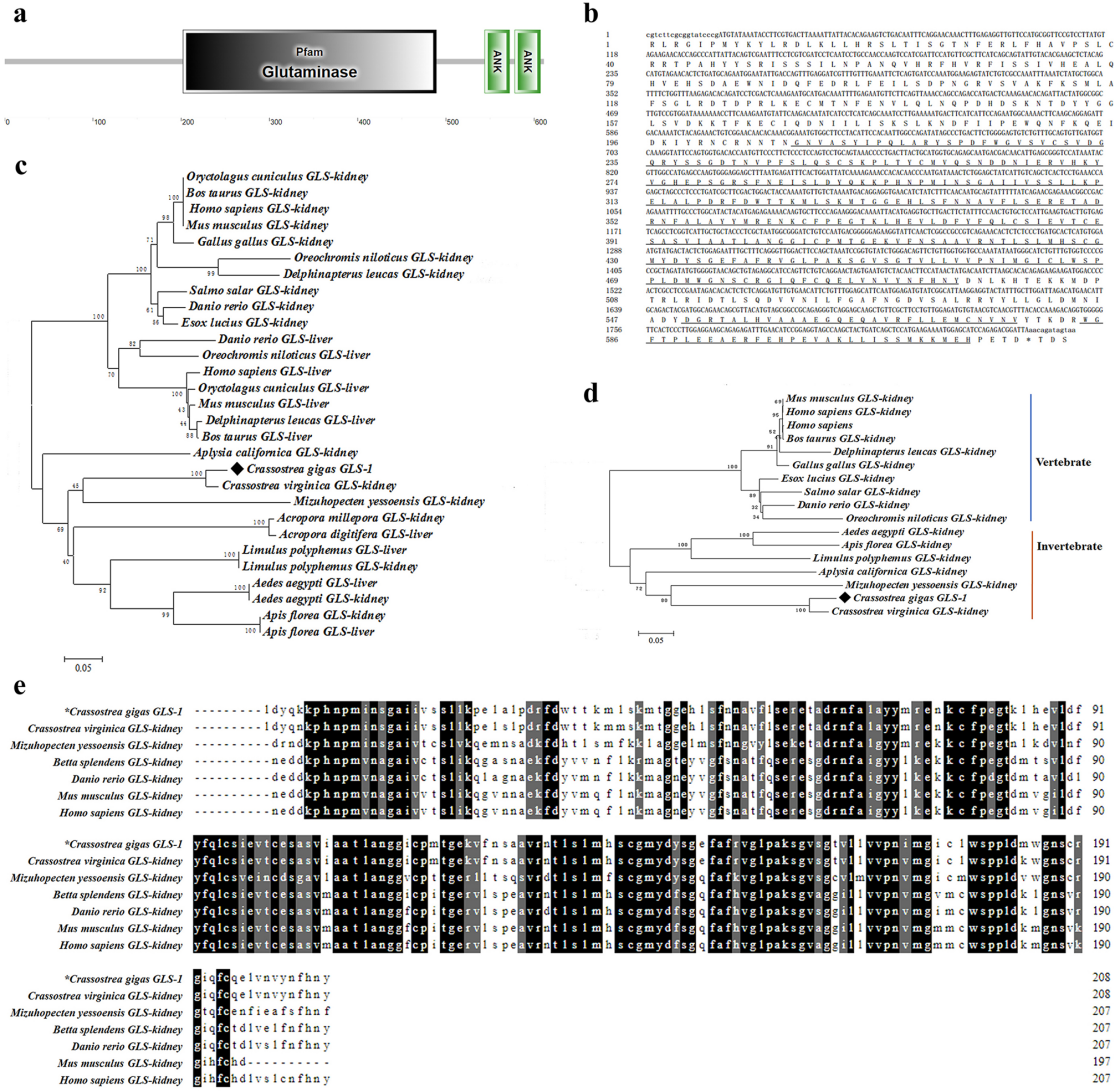
The sequence characteristics of *Cg*GLS-1. (**a**) The structure of *Cg*GLS-1 predicted by SMART. *Cg*GLS-1 protein contains a Glutaminase domain (202–489 aa), and two ANK domains (544–607 aa). (**b**) The ORF of *Cg*GLS-1. The GLS domain and ANK domains are underlined. (**c**,**d**) The Neighbor-joining (NJ) phylogeny tree of GLS-1 from various species including vertebrates and invertebrates. The information of sequences used for the *Cg*GLS-1 phylogenetic analysis was shown in Table 2. The bootstrap value is displayed by the numbers at the forks. (**e**) Multiple sequences alignment of *Cg*GLS-1 with other GLS from vertebrates and invertebrates. The black shadow region indicates positions where all sequences share the same amino acid residue. Similar amino acids are shaded in grey. The information of sequences used for the *Cg*GLS-1 alignment is shown in Table 2.

The open reading frame (ORF) of *Cg*CLS-1 was of 1836 bp, encoding a putative peptide of 611 amino acids (Fig. 1b) with a molecular mass of 69.64 kDa and theoretical isoelectric point of 6.14. SMART analysis revealed that there was one Glutaminase domain (202–489 aa) and two ankyrin (ANK) domains (544–607 aa) in the deduced amino acid sequences of *Cg*GLS-1 protein (Fig. 1a).

**Table 2:** Sequences used for the *Cg*GLS-1 alignment and phylogenetic analysis.

Accession number	Gene name	Organism
XP_011435002.1	PREDICTED: glutaminase kidney isoform, mitochondrial isoform X1	*Crassostrea gigas*
NP_001074550.1	glutaminase kidney isoform, mitochondrial isoform 1	*Mus musculus*
XP_022300661.1	glutaminase kidney isoform, mitochondrial-like isoform X1	*Crassostrea virginica*
XP_021377493.1	glutaminase kidney isoform, mitochondrial-like isoform X1	*Mizuhopecten yessoensis*
XP_022245497.1	glutaminase liver isoform, mitochondrial-like isoform X2	*Limulus polyphemus*
XP_022245498.1	glutaminase kidney isoform, mitochondrial-like isoform X3	*Limulus polyphemus*
XP_021698607.1	glutaminase liver isoform, mitochondrial isoform X1	*Aedes aegypti*
XP_021698608.1	glutaminase kidney isoform, mitochondrial isoform X3	*Aedes aegypti*
XP_012940993.1	PREDICTED: glutaminase kidney isoform, mitochondrial-like	*Aplysia californica*
XP_005167956.1	glutaminase kidney isoform, mitochondrial isoform X2	*Danio rerio*
XP_001345099.5	glutaminase liver isoform, mitochondrial isoform X2	*Danio rerio*
NP_055720.3	glutaminase kidney isoform, mitochondrial isoform 1 precursor	*Homo sapiens*
NP_037399.2	glutaminase liver isoform, mitochondrial isoform 1 precursor	*Homo sapiens*
NP_001028436.2	glutaminase liver isoform, mitochondrial isoform 1 precursor	*Mus musculus*
XP_010889082.1	glutaminase kidney isoform, mitochondrial isoform X1	*Esox lucius*
XP_025762872.1	glutaminase liver isoform, mitochondrial isoform X1	*Oreochromis niloticus*
XP_005463410.1	glutaminase kidney isoform, mitochondrial isoform X4	*Oreochromis niloticus*
XP_012347237.1	glutaminase kidney isoform, mitochondrial isoform X4	*Apis florea*
XP_012347229.1	glutaminase liver isoform, mitochondrial isoform X3	*Apis florea*
XP_029179824.1	glutaminase kidney isoform, mitochondrial-like isoform X2	*Acropora millepora*
XP_015764319.1	PREDICTED: glutaminase liver isoform, mitochondrial-like isoform X1	*Acropora digitifera*
NP_001026419.1	glutaminase kidney isoform, mitochondrial precursor	*Gallus gallus*
XP_014007049.1	PREDICTED: glutaminase kidney isoform, mitochondrial-like isoform X1	*Salmo salar*
XP_002711127.1	PREDICTED: glutaminase liver isoform, mitochondrial isoform X1	*Oryctolagus cuniculus*
XP_002712390.1	PREDICTED: glutaminase kidney isoform, mitochondrial	*Oryctolagus cuniculus*
XP_022422980.1	glutaminase kidney isoform, mitochondrial isoform X2	*Delphinapterus leucas*
XP_022427521.1	glutaminase liver isoform, mitochondrial isoform X1	*Delphinapterus leucas*
XP_005202636.1	glutaminase kidney isoform, mitochondrial isoform X1	*Bos taurus*
XP_005206712.1	glutaminase liver isoform, mitochondrial isoform X1	*Bos taurus*

Two phylogenic trees were constructed based on the amino acid sequences of *Cg*GLS-1 and GLSs (both liver-type and kidney-type GLS) from other species (Fig. 1c). The vertebrate’s kidney-type GLS and liver-type GLS were clustered into distinct branches in the phylogenic trees. But there was no obvious differentiation between kidney-type GLS and liver-type GLS in invertebrates (Fig. 1c). *Cg*GLS-1was clustered with the kidney-type GLS of *C. virginica* and *Mizuhopecten yessoensis* (Fig. 1c), indicating that it exhibited higher homology to kidney-type GLS. Another phylogenic tree based on the amino acid sequences of *Cg*GLS-1 and kidney-type GLS from other species was constructed to investigate the evolutionary status of *Cg*GLS-1 (Fig. 1d). GLSs from vertebrates and invertebrates were separated clearly into different branches. *Cg*GLS-1 was first clustered with kidney-type GLS of *C. virginica* and formed a molluscan branch with those from other molluscs (Fig. 1d).

The deduced amino acid sequence of Glutaminase domain in *Cg*GLS-1 shared high homology with that of other GLSs, such as 97.6% identity with Glutaminase domain of *C. virginica* kidney-type GLS, 68.3% identity with Glutaminase domain of *M. yessoensis* kidney-type GLS, and 65.4% identity with Glutaminase domain of *Homo sapiens* kidney-type GLS (Fig. 1e).

### Activity of recombinant protein of *Cg*GLS-1 and the specificity of its polyclonal antibody

**Figure 2:**
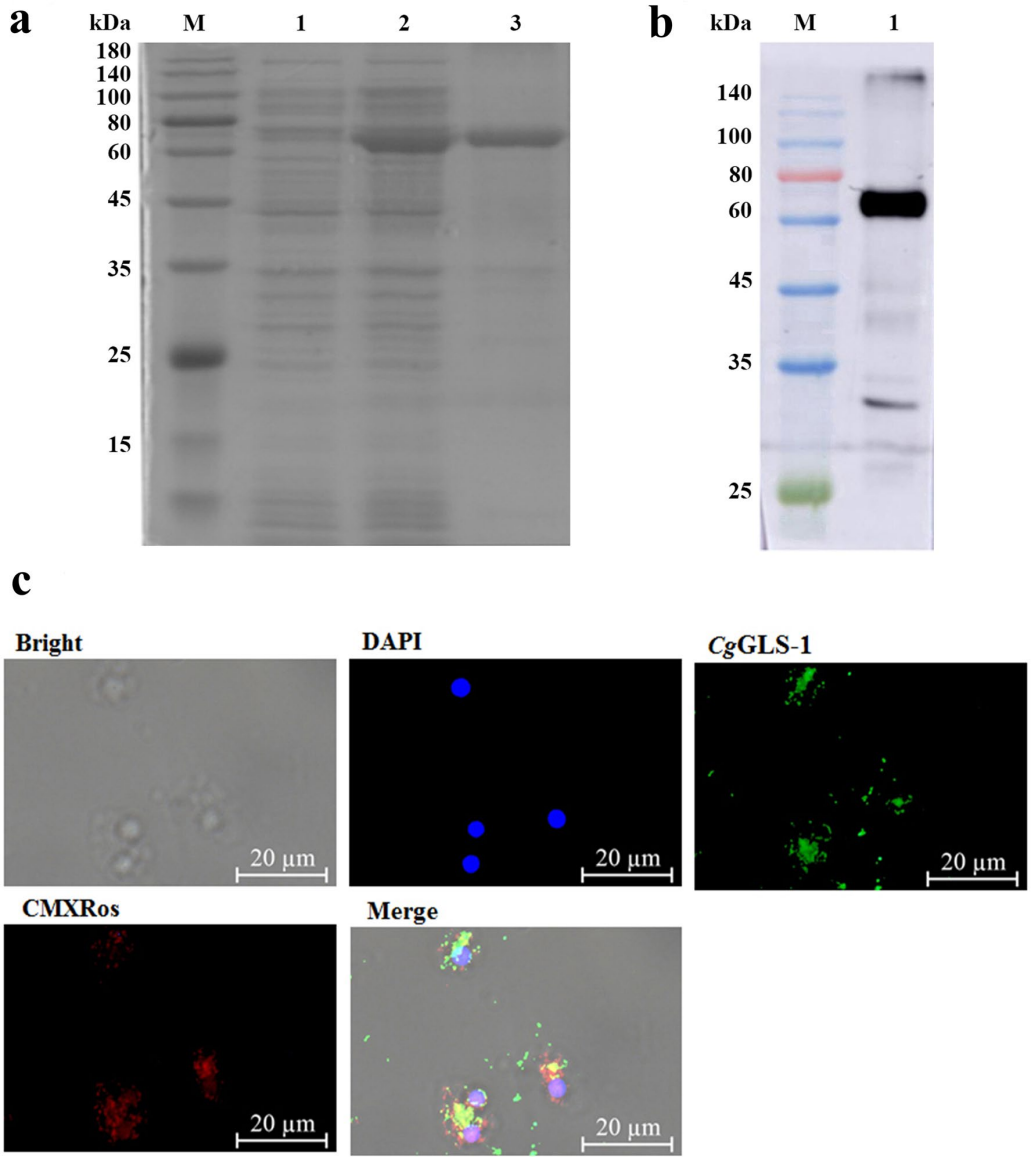
SDS-PAGE and Western blot analysis of recombinant *Cg*GLS-1, and subcellular localization of *Cg*GLS-1 in *C. gigas* hemocyte. (**a**) SDS-PAGE of analysis r*Cg*GLS-1. Lane M: protein molecular standard (kDa); Lane 1: negative control for r*Cg*GLS-1 (without induction); Lane 2: induced r*Cg*GLS-1; Lane 3: purified r*Cg*GLS-1. (**b**) Western blot analysis of *anti*-r*Cg*GLS-1. Lane M: protein molecular standard (kDa); Lane 1: Western blot based on the sample of Line 3. (**c**) Subcellular localization of *Cg*GLS-1 in *C. gigas* hemocyte. Nuclei staining with DAPI are shown in blue; *anti*-r*Cg*GLS-1 conjugated to Alexafluor 488 is shown in green; the cell mitochondria are stained by Mito-Tracker Red CMXRos and is shown in red. The scale bar is 20 μm. (Full-length blots/gels are presented in Supplementary Fig. 1 and 2).

The recombinant plasmid pET-30a-*Cg*GLS-1 was transformed into *E. coli* BL21 (DE3). After IPTG induction, the whole cell lysate of positive clone was analyzed by SDS-PAGE, and a distinct band with a molecular weight of ~ 69 kDa was observed (Fig. 2a), which was consistent with the predicted molecular weight of *Cg*GLS-1. The activity of r*Cg*GLS-1 was determined by GLS kit as described above. The content of the purified r*Cg*GLS-1 was determined to be 281 μg/mL. The enzyme activity of r*Cg*GLS-1 was estimated to be 1.705 U/mg, and the enzyme activity of positive control was estimated to be 5.653 U/mL. While the enzyme activity of negative control was nearly undetectable.

The purified r*Cg*GLS-1 protein was utilized to prepare polyclonal antibody. The antibody specificity was tested by Western blot and a clear band about 69 kDa was revealed, which was coincident with the predicted molecular mass of r*Cg*GLS-1 (Fig. 2b). No visible band was detected in the group of the mouse pre-immune serum (data not shown).

### Localization of *Cg*GLS-1 in oyster hemocytes

Subcellular localization of *Cg*GLS-1 in hemocytes was determined by immunohistochemistry assay. In oyster hemocytes, the nucleus was stained by DAPI in blue and the immunoreactive area for *Cg*GLS-1 labeled by Alexa Fluor 488-conjugated antibody was in green. Mitochondria was stained by Mito-Tracker Red CMXRos with the color of red. The positive signal of *Cg*GLS-1 was mainly distributed in the cytoplasm with obvious co-location of mitochondria in hemocytes (Fig. 2c).

### Distribution of *Cg*GLS-1 mRNA transcripts in different oyster tissues

**Figure 3:**
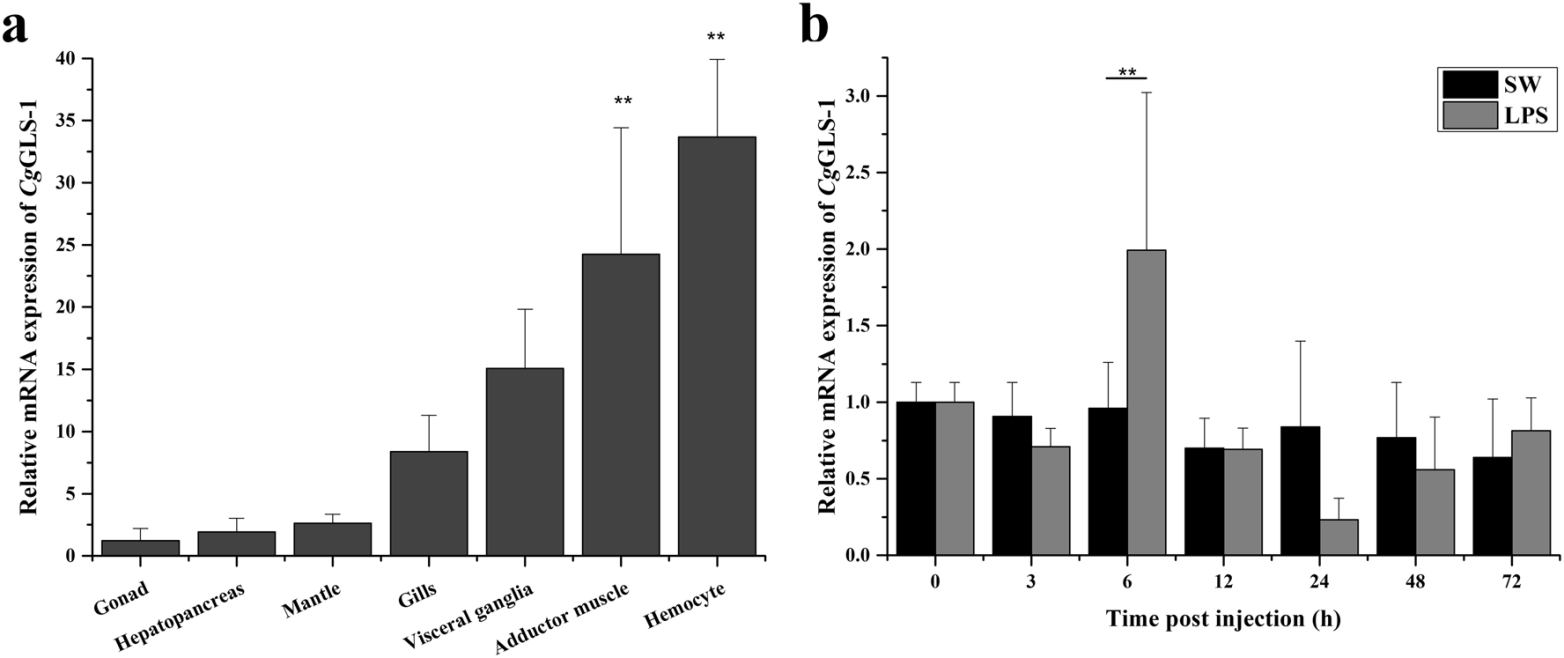
The mRNA expression profile of *Cg*GLS-1. (**a**) The relative mRNA expression levels of *Cg*GLS-1 mRNA in different tissues detected by qRT-PCR. Data were represented as the ratio of *Cg*GLS-1 mRNA level to that of gonad and normalized to that of *Cg*EF. (**b**) The mRNA expression patterns of *Cg*GLS-1 in oyster hemocytes after LPS stimulation. Data were represented as the ratio of *Cg*GLS-1 mRNA level to that of 0 h and normalized to that of *Cg*EF. Comparison of the level of *Cg*GLS-1 mRNA (relative to *Cg*EF) was normalized to 0 h. Each value is shown as mean ± S.D. (N = 3). Asterisks indicate significant differences. (**p < 0.01).

Quantitative real-time PCR was employed to investigate the expression level of *Cg*GLS-1 mRNA in different tissues with *Cg*EF as internal control. *Cg*GLS-1 specific primers P8 and P9 (Table 1) were used to amplify a fragment of 176 bp. For *Cg*GLS-1 and *Cg*EF genes, there was only one peak at the corresponding melting temperature in the dissociation curve analysis, indicating that the target sequence was specifically amplified (data not shown). The *Cg*GLS-1 transcripts were detectable in all the tested tissues including gonad, hepatopancreas, mantle, gill, visceral ganglia, adductor muscle, and hemocytes. The highest expression level of *Cg*GLS-1 mRNA was detected in hemocytes, which was 33.67-fold (*p* < 0.01) higher than that in gonad (Fig. 3a). The mRNA expression level of *Cg*GLS-1 in adductor muscle and visceral ganglia was significantly 24.25- and 15.08-fold (*p* < 0.01) higher than that in gonad, respectively. There was no significant difference of *Cg*GLS-1 mRNA expression in hepatopancreas, mantle and gill compared to that in gonad (Fig. 3a).

### The mRNA expression of *Cg*GLS-1 in hemocytes after LPS stimulation

The expression of *Cg*GLS-1 mRNA in hemocytes after LPS stimulation was quantified by quantitative real-time PCR to investigate its possible functions in immune defense. The mRNA expression level of *Cg*GLS-1 in hemocytes was significantly increased and reached the peak level (2.07-fold of that in SW group, *p* < 0.01) at 6 h, then recovered to the normal level at 12 ~ 72 h. No significant change of *Cg*GLS-1 mRNA expression in hemocytes was observed in the control (SW) group (Fig. 3b).

### The change of Glu concentration in serum after BPTES and LPS treatments

**Figure 4:**
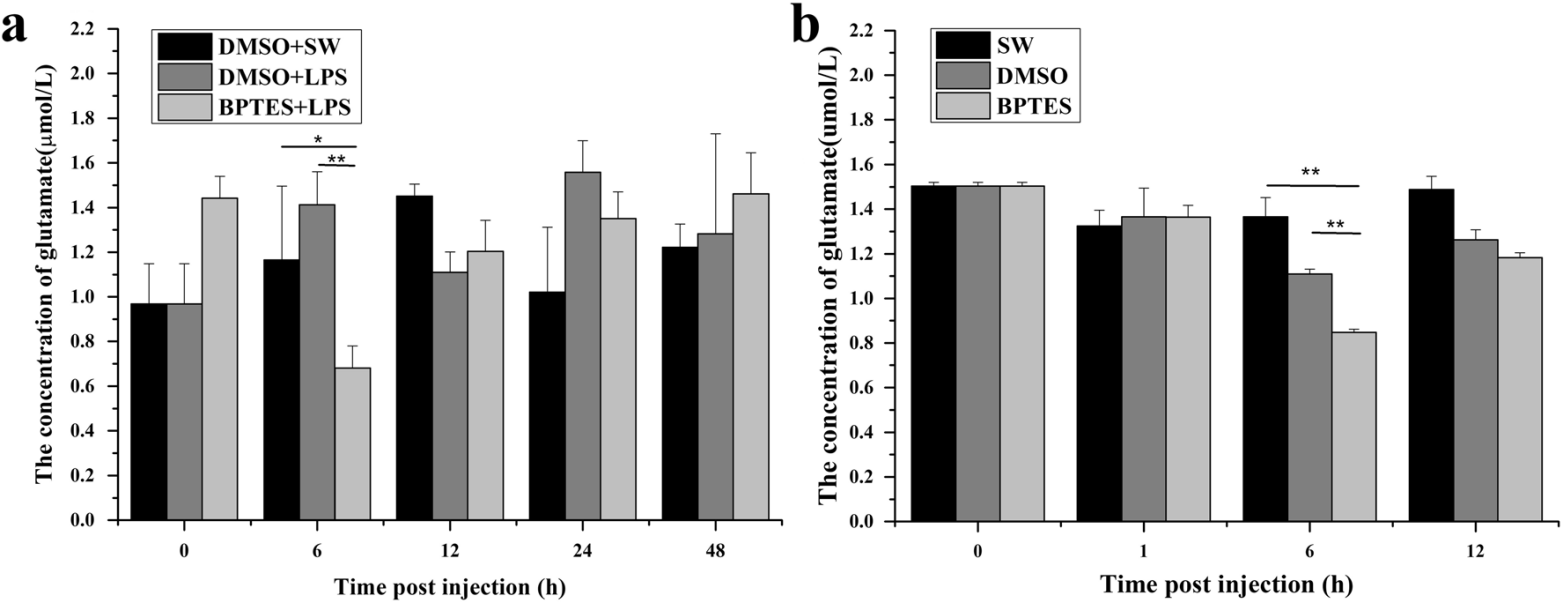
The content of Glu in serum of oysters after BPTES and LPS treatments. (**a**) The content of Glu after BPTES injection. (**b**) The content of Glu after BPTES and LPS stimulation. Vertical bars represent the mean ± S.D. (N = 3). Asterisks indicate significant differences. (*p < 0.05; **p < 0.01).

The concentration of Glu in oyster serum after BPTES and LPS treatments was quantified by Glutamate ELISA Kit. In BPTES group, the content of Glu decreased significantly (0.8468 μmol/L, 0.62-fold of that in SW group, *p* < 0.01) at 6 h after BPTES injection (Fig. 4a). After the oysters were pre-treated with an injection of BPTES and then stimulated with LPS (BPTES + LPS group), the concentration of Glu decreased to 0.6812 μmol/L at 6 h after LPS injection, which was significantly lower than that in DMSO + LPS group (1.4122 μmol/L, *p* < 0.01) (Fig. 4b). No significant change of Glu concentration was observed in SW and DMSO groups.

### The mRNA expression of *Cg*IL17-5, *Cg*TNF-1, *Cg*mGluR6 and *Cg*AP-1 in hemocytes after BPTES and LPS treatments

**Figure 5:**
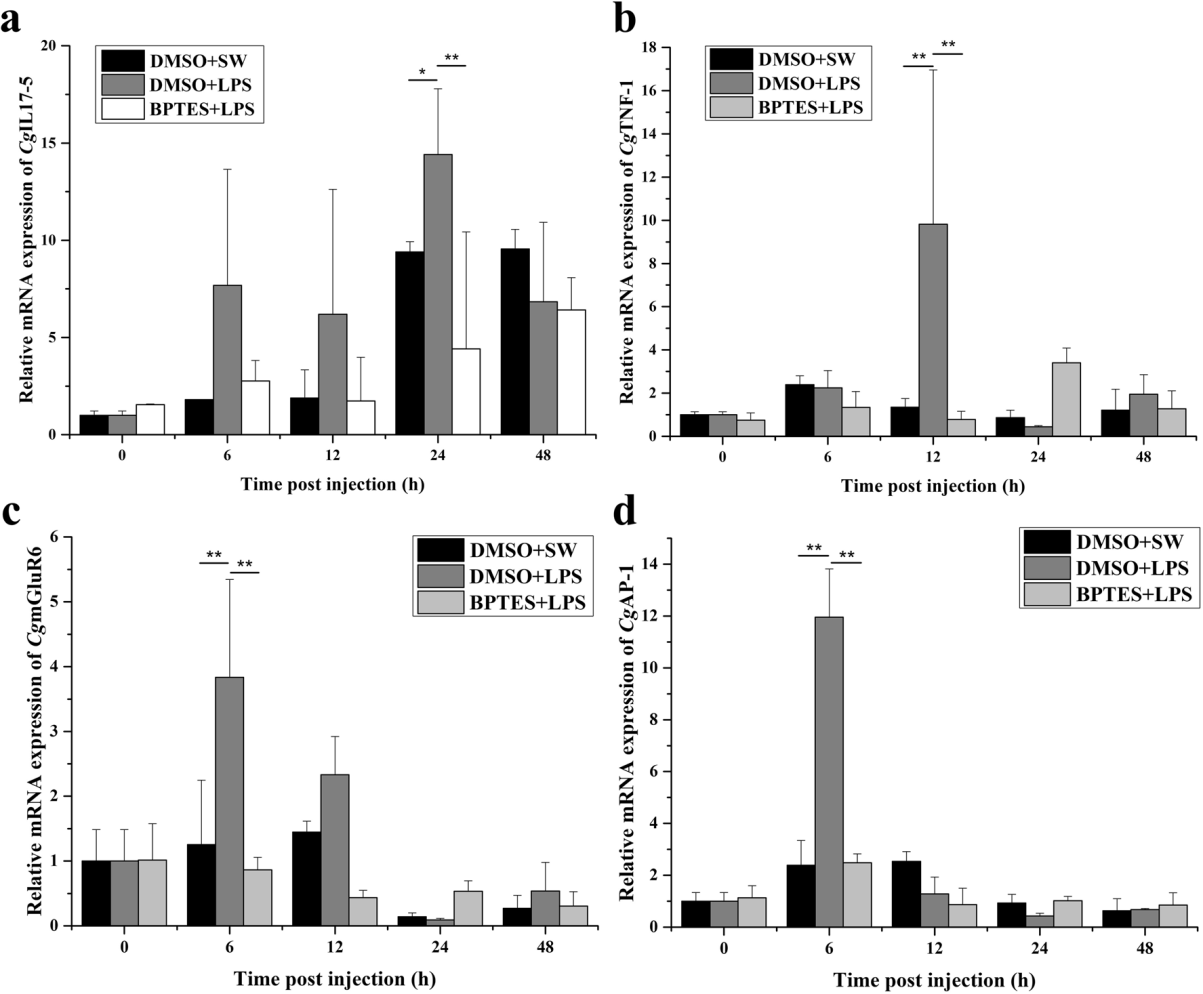
The temporal expression of *Cg*IL17-5 (**a**), *Cg*TNF-1 (**b**), *Cg*mGluR6 (**c**) and *Cg*AP-1 (**d**) after BPTES and LPS treatments. Each group value is shown as mean ± S.D. (N = 3). Asterisks indicate significant differences. (*p < 0.05; **p < 0.01).

The mRNA expression of *Cg*IL17-5, *Cg*TNF-1, *Cg*mGluR6 and *Cg*AP-1 was quantified by quantitative real-time PCR after BPTES and LPS treatments. In DMSO + LPS group, the expression of *Cg*IL17-5 increased (1.53-fold of that in DMSO + SW group, *p* < 0.05) at 24 h after LPS injection (Fig. 5a), while the mRNA transcripts of *Cg*TNF-1 increased significantly (7.25-fold of that in DMSO + SW group, *p* < 0.01) at 12 h (Fig. 5b). Also, in DMSO + LPS group, the expression of *Cg*mGluR6 and *Cg*AP-1 increased significantly at 6 h (5.00- and 3.05-fold of that in DMSO + SW group, respectively, *p* < 0.01) (Fig. 5c,d). However, the increase of the *Cg*IL17-5, *Cg*TNF-1, *Cg*mGluR6 and *Cg*AP-1 mRNA expression was reverted when the oysters were treated with GLS inhibitor BPTES before LPS stimulation. Specifically, the expression of *Cg*IL17-5 in BPTES + LPS group decreased significantly (0.47-fold of that in DMSO + SW group, *p* < 0.01) at 24 h after LPS injection. And the expression of *Cg*TNF-1 in BPTES + LPS group decreased significantly (0.57-fold of that in DMSO + SW group, *p* < 0.01) at 12 h after LPS injection (Fig. 5a,b). While the expression of *Cg*mGluR6 and *Cg*AP-1 in BPTES + LPS group decreased significantly at 6 h (0.69- and 1.04-fold of that in DMSO + SW group, respectively, *p* < 0.01) (Fig. 5c,d).

### The apoptosis rates of hemocytes and mRNA expression of *Cg*Caspase3 in hemocytes after BPTES and LPS treatments

**Figure 6:**
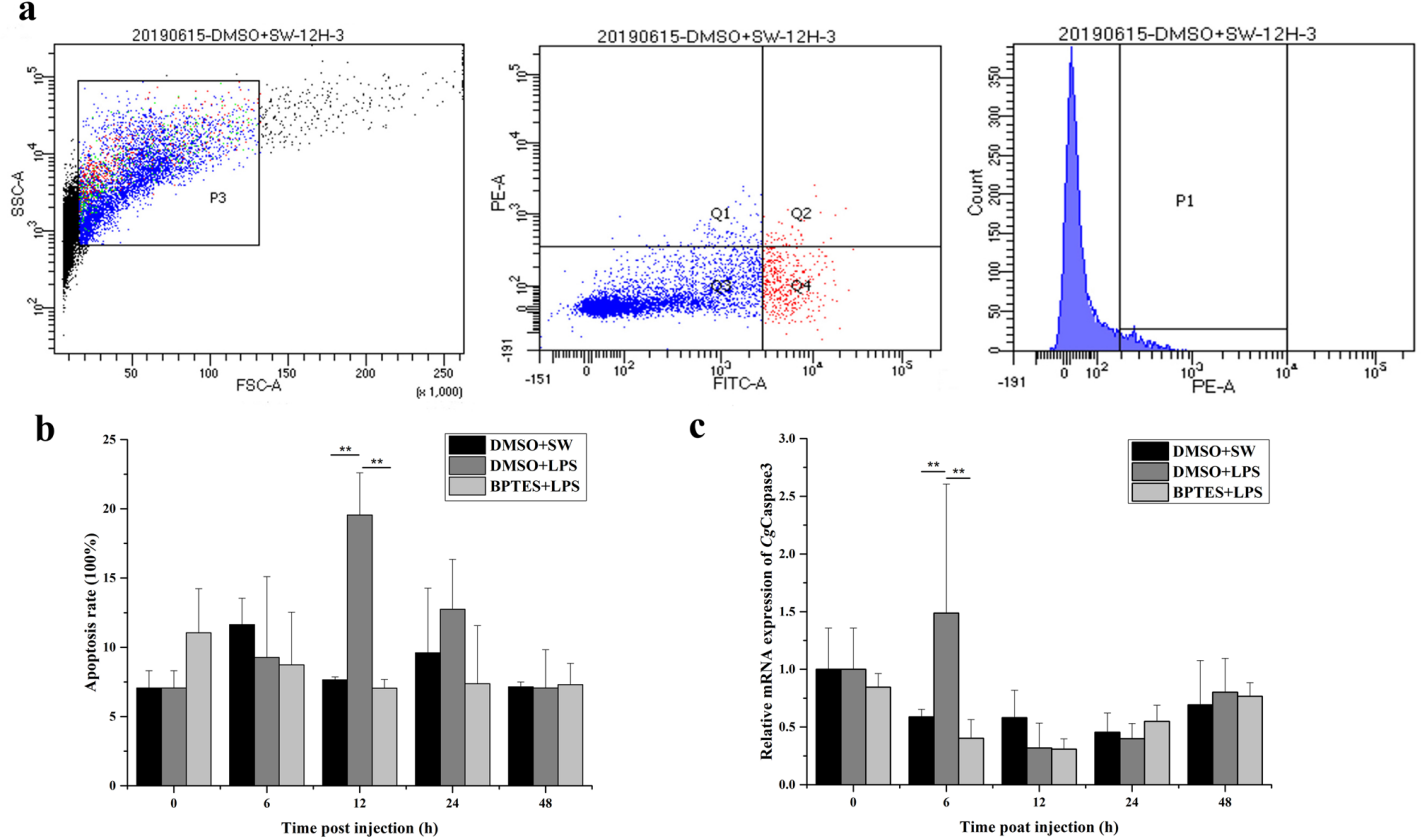
The apoptosis rate of hemocytes and expression of *Cg*Caspase3 after in vivo treatment of oysters with BPTES and LPS. (**a**) Determination of hemocyte apoptosis index by flow cytometry. (**b**) The apoptosis index of hemocyte after BPTES and LPS injection. Quantitative graph of the data in hemocytes of different treatments, indicating the changes of the proportion of early apoptotic cells. (**c**) Relative expression of *Cg*Caspase3 gene in oyster hemocytes after BPTES and LPS injection. Each group value is shown as mean ± S.D. (N = 3). Asterisks indicate significant differences. (**p < 0.01).

The mRNA expression level of *Cg*Caspase3 was quantified by quantitative real-time PCR. The apoptosis rates of hemocytes were detected by FCM. Forward scatter (FSC) and side scatter (SSC) parameters were used to indicate cell size and granularity. The early apoptosis rates were equal to the percentage of hemocytes with Annexin V positive and PI negative hemocytes (Fig. 6a). In DMSO + LPS group, the mRNA expression level of *Cg*Caspase3 increased significantly and reached the peak level (2.53-fold of that in DMSO + SW group, *p* < 0.01) at 6 h after LPS injection (Fig. 6c), and the apoptosis rate of hemocytes also increased significantly, which was 19.55% (2.55-fold of that in DMSO + SW group, *p* < 0.01) at 12 h after LPS injection. However, the apoptosis rate in BPTES + LPS group decreased significantly (7.05%, 0.92-fold of that in DMSO + SW group, *p* < 0.01) at 12 h after LPS injection (Fig. 6b). The up-regulation of *Cg*Caspase3 mRNA was also reverted when the oysters were pretreated with BPTES. The mRNA transcripts of *Cg*Caspase3 in BPTES + LPS group decreased significantly (0.68-fold of that in DMSO + SW group, *p* < 0.01) at 6 h after LPS injection (Fig. 6c).

## Discussion

GLS is an essential component of glutamatergic system, which synthesizes Glu from glutamine. The delicate balance of this system is very important for maintaining normal physiological homeostasis, and any changes of Glu level in the plasma are associated with diseases. Previous study has demonstrated that *C. gigas* have evolved with the capability to adjust the immune response level by regulating the balance between Glu and GABA^28^, and in the early stage of immune response, the secretion of Glu is activated to eliminate pathogens quickly, while the synthesis of GABA is triggered to avoid excess reactions in the late stage of the immune response^35,52^. In the present study, the immunomodulatory effect of glutamatergic system in *C. gigas* was further explored to provide helpful evidences to better understand the involvement of glutamatergic system in the immune defense of marine molluscs.

Glutaminases have been relatively well studied in vertebrates, which function as an amidohydrolase enzyme to hydrolyze glutamine to glutamate, and they are characterized as two different isoforms, the kidney-type GLS and liver-type GLS. In the present study, a glutaminase gene was identified in *C. gigas*, which contained a Glutaminase domain (202–489 aa) and two ANK domains (544–607 aa). The ANK domain in GLS mediates the interaction of GLS with other proteins, and the Glutaminase domain catalyzes the hydrolysis of glutamine to Glu and ammonia^53^. The deduced amino acid sequence of Glutaminase domain in *Cg*GLS-1 shared high homology with that of Glutaminase domain in kidney-type glutaminase, such as 97.6% identity with that of *C. virginica* kidney-type glutaminase, and 65.4% identity with that of *Homo sapiens* kidney-type glutaminase. In the phylogenic tree, the *Cg*GLS-1 was first clustered with kidney-type GLS of *C. virginica* and *M. yessoensis*, and then grouped into molluscan branch with GLS from other molluscs, indicating that *Cg*GLS-1 is evolutionarily related to kidney-type GLS in invertebrates. Recently, a highly conserved kidney-type GLS were also identified from bony fish *Siniperca chuatsi*^54^, suggesting kidney-type GLS was evolutionarily conserved. All the above results suggested that *Cg*GLS was a novel member of glutaminases in molluscs, which most likely belongs to the kidney-type GLS family.

Increasing evidences have demonstrated that GLS plays important roles in some physiological processes in mammals, such as the ammonia and urea genesis as well as the synthesis of Glu. In order to understand the function of *Cg*GLS-1 on the synthesis of glutamate and its potential physiological roles, the enzyme activity of r*Cg*GLS-1 and the expression of *Cg*GLS-1 in different tissues were determined. The enzyme activity of r*Cg*GLS-1 was estimated to be 1.705 U/mg. Moreover, the content of Glu in the serum decreased significantly (0.8468 μmol/L, 0.62-fold of that in SW group, *p* < 0.01) at 6 h after the injection of inhibitor of BPTES, indicating that it was able to catalyze the hydrolysis of glutamine to Glu and ammonia. Previous studies in vertebrates have found that GLSs are widely expressed in many tissues including kidney, adrenal, small intestine, brain, duodenum, even skeletal and cardiac muscle, and so on^3^. In the present study, *Cg*GLS-1 mRNA was constitutively expressed in all the tested tissues, including mantle, gonad, gills, adductor muscle, hemocytes, visceral ganglia and hepatopancreas, suggesting that Glu might be synthesized in different tissues of oysters. The highest expression of *Cg*GLS was observed in hemocytes of oysters. Since hemocytes can be present in all the tissues, it could not exclude that the expression of GLS in other tissues came from infiltrated hemocytes or at least partially. In mammals, kidney-type glutaminase is always detected in neuronal mitochondria, while the liver-type glutaminase is reported to have an extramitochondrial location in neuronal nuclei of rat and monkey brain^55–57^. The mitochondrial location of kidney-type glutaminase in astrocytes is suggested to be a control mechanism allowing broad and fine tuning of Glu production depending on their energetic needs and/or synaptic activity^23^. In the present study, *Cg*GLS-1 was mainly distributed over the cytoplasm with a co-location of mitochondria in oyster hemocytes. The result was consistent with the previous reports about vertebrate kidney-type glutaminases that were predominantly localized to the inner mitochondrial membrane. These results suggested that *Cg*GLS-1 functioned as a kidney-type GLS for the synthesis of Glu, which was located on the mitochondria of the cells of *C. gigas*.

The glutaminase expressed in the immune cells of vertebrates is found to exert an important role in immune reactions by its production Glu^17,18,58^. In the present study, the highest expression level of *Cg*GLS-1 was observed in hemocytes, compared to the tested tissues, which was dramatically 33.67-fold (*p* < 0.01) higher than that in gonad. Hemocytes are considered as important components in the immune response of molluscs^30,59^. The high expression of *Cg*GLS-1 in hemocytes might indicate its vital role in the immune response of oyster. Moreover, the mRNA expression of the *Cg*GLS-1 in hemocytes was significantly up-regulated at 6 h after LPS stimulation. Similarly, the increased glutaminase expression after LPS injection was also found in other species such as rainbow trout, which was deemed to supply fuels and signals in the culture media in response to LPS challenge^60^. These results collectively indicated that *Cg*GLS-1 might play an import role on anti-bacterial immunity of oyster.

It is reported that Glu modulates the secretion of several cytokines, playing a regulatory role on the immune response of vertebrates. For example, Glu released by dendritic cells (DC) cells impairs IL-6 production through mGlu5R expressed in resting human T cells^18^. In the present study, the content of Glu decreased significantly after the injection of a specific inhibitor of kidney-type glutaminase (BPTES), indicating that the generation of Glu was suppressed after the injection of the specific inhibitor of kidney-type glutaminase. Moreover, the expression of *Cg*mGluR6 was significantly increased after LPS stimulation, and this up-regulation was reverted when the oysters were pretreated with BPTES. Glu receptors exist in immune cells and induce various immune reactions such as T cell adhesion and chemotactic migration, cytokine secretion in vertebrates^13,14^. In invertebrates, some Glu receptors have also been identified in insects and nematodes, which can mediate neurotransmission at synapses^23,61^. The above results indicated that *Cg*GLS-1 regulated the production of Glu in oyster hemocytes and might play an immunomodulatory role through Glu receptor *Cg*mGluR6. Glu couples to the extracellular signal-regulated kinases (ERK)-pathway to enhance the secretion of IL-6, TNF-α, Th1 cytokines (IL-2 and IFN-γ), IL-10 via mGlu1R which expresses upon T cell activation^13,14,62^. Recently, some cytokines, such as IL, TNF, and IFN family members have been identified in oysters^63^. In the present study, the expression level of *Cg*AP-1, *Cg*IL17-5, and *Cg*TNF-1 mRNA increased significantly after LPS stimulation, while the up-regulation were reverted when the oysters were pretreated with BPTES, suggesting that *Cg*GLS-1 was able to modulate the production of *Cg*IL17-5, and *Cg*TNF-1 in oyster hemocytes. *Cg*IL17-5 was inferred to activate the transcription factors NF-kB, CREB and ATF-1 and play an important role in the immune defense^64^. Similar to the observation in vertebrates that the transcription factor AP-1 can regulate the production of cytokines^65^, *Cg*AP-1 was also found to regulate the expression of *Cg*IL17-5 in oysters^41^. These results suggested that *Cg*GLS-1 can be involved in regulating the expression of *Cg*IL17-5 through transcription factor *Cg*AP-1. It is reported that *Cg*TNF-1 not only regulates phagocytosis of hemocytes, but also modulates PO, lysozyme, and anti-bacterial activities^66^. *Cg*TNF-1 can also trigger the activation of transcription factors NF-κB and HSF through the activation of MAPK signal pathway, and then regulate the apoptosis^40^. These results suggested that *Cg*GLS-1 regulated the secretion of *Cg*IL17-5 and *Cg*TNF-1 possibly via the Glu receptor *Cg*mGluR6, but the detailed mechanism and the associated pathways in oysters still need further investigation.

Apoptosis is the process of programmed cell death as a defense mechanism in immune reactions. Glu is found to induce cell death of primary cultures of rat cortical cells and involved in the regulation of caspase-3 protease^67^. In the present study, the mRNA transcripts of *Cg*Caspase3 increased significantly at 6 h (*p* < 0.01) after LPS stimulation. However, the up-regulation of the mRNA transcripts of *Cg*Caspase3 after LPS stimulation was reverted when the oysters were pretreated with BPTES. These results indicated that *Cg*GLS-1 could regulate the expression of caspase in oysters. In human, the activation of NMDA type iGluRs induced by Glu caused the increases of cytosolic Ca^2+^ concentration and activation of Ca^2+^-dependent protein kinase C that resulted in the increase of ROS levels, and eventually stimulated either necrotic cell death, or apoptotic cell death via activation of caspase-3^12,68^. In oyster, *Cg*Caspase3 exhibited caspase activity and could induce cell apoptosis *in vivo*^69^. In the present study, the apoptosis rate of hemocytes increased significantly at 12 and 24 h after LPS stimulation. But it decreased significantly at 12 and 24 h after LPS stimulation when the oysters were pretreated with an injection of BPTES. The present results suggested that *Cg*GLS-1 was able to regulate the expression of *Cg*Caspase3 as well as the apoptosis of hemocytes against invading bacteria.

## Conclusion

In conclusion, a homologue of GLS, *Cg*GLS-1, was identified in *C. gigas*, which shared higher similarity with kidney-type GLS of vertebrates and was able to catalyze the hydrolysis of glutamine to Glu. *Cg*GLS-1 was involved in the anti-bacterial immune response of *C. gigas* by regulating the expression of *Cg*AP-1, *Cg*IL17-5 and *Cg*TNF-1, and the apoptosis of hemocytes, via mediating the production of Glu as well as the expression of Glu receptor *Cg*mGluR6 in the hemocytes. The results provided insights into the function of glutamate energic system in the immune defense of marine molluscs.

## Supplementary Information

Supplementary figures.

## References

[1] Marquez J, La Oliva ARLD, Mates JM, Segura JA, Alonso FJ (2006). Glutaminase: a multifaceted protein not only involved in generating glutamate. Neurochem. Int..

[2] Aledo JC, Gomezfabre PM, Olalla L, Marquez J (2000). Identification of two human glutaminase loci and tissue-specific expression of the two related genes. Mamm. Genome.

[3] Curthoys NP, Watford M (1995). Regulation of glutaminase activity and glutamine metabolism. Annu. Rev. Nutr..

[4] Watford M, Smith EM, Erbelding EJ (1984). The regulation of phosphate-activated glutaminase activity and glutamine metabolism in the streptozotocin-diabetic rat. Biochem. J..

[5] Rohde T, Maclean DA, Pedersen BK (1996). Glutamine, lymphocyte proliferation and cytokine production. Scand. J. Immunol..

[6] Welbourne T, Nissim I (2001). Regulation of mitochondrial glutamine/glutamate metabolism by glutamate transport: studies with (15)N. Am. J. Physiol. Cell Physiol..

[7] Kovacevic Z, Mcgivan JD (1983). Mitochondrial metabolism of glutamine and glutamate and its physiological significance. Physiol. Rev..

[8] Neu J, Shenoy V, Chakrabarti R (1996). Glutamine nutrition and metabolism: where do we go from here?. Faseb J..

[9] Curi R (2016). Regulatory principles in metabolism–then and now. Biochem. J..

[10] Krebs HA (1935). Metabolism of amino-acids: The synthesis of glutamine from glutamic acid and ammonia, and the enzymic hydrolysis of glutamine in animal tissues. Biochem. J..

[11] Pacheco R, Gallart T, Lluis C, Franco R (2007). Role of glutamate on T-cell mediated immunity. J. Neuroimmunol..

[12] Boldyrev AA, Carpenter DO, Johnson P (2005). Emerging evidence for a similar role of glutamate receptors in the nervous and immune systems. J. Neurochem..

[13] Ganor Y, Besser MJ, Benzakay N, Unger T, Levite M (2003). Human T cells express a functional ionotropic glutamate receptor GluR3, and glutamate by itself triggers integrin-mediated adhesion to laminin and fibronectin and chemotactic migration. J. Immunol..

[14] Dutta G, Goswami AR, Ghosh T (2013). Effects of stimulation of glutamate receptors in medial septum on some immune responses in rats. Brain Res..

[15] Green DR, Droin NM, Pinkoski MJ (2003). Activation-induced cell death in T cells. Immunol. Rev..

[16] Ishiuchi S (2002). Blockage of Ca ^2+^ -permeable AMPA receptors suppresses migration and induces apoptosis in human glioblastoma cells. Nat. Med..

[17] Lombardi G (2004). Glutamate modulation of human lymphocyte growth: in vitro studies. Biochem. Biophys. Res. Commun..

[18] Pacheco R (2006). Glutamate released by dendritic cells as a novel modulator of T cell activation. J. Immunol..

[19] Gou Z, Wang X, Wang W (2013). Evolution of neurotransmitter gamma-aminobutyric acid, glutamate and their receptors. Zool. Res..

[20] Kolodziejczyk A, Sun X, Meinertzhagen IA, Nässel DR (2008). Glutamate, GABA and acetylcholine signaling components in the lamina of the Drosophila visual system. PLoS ONE.

[21] Elliott GR, Leys SP (2010). Evidence for glutamate, GABA and NO in coordinating behaviour in the sponge, *Ephydatia muelleri* (Demospongiae, Spongillidae). J. Exp. Biol..

[22] Alberstein R, Grey R, Zimmet A, Simmons DK, Mayer ML (2015). Glycine activated ion channel subunits encoded by ctenophore glutamate receptor genes. Proc. Natl. Acad. Sci. U. S. A..

[23] Hart AC, Sims S, Kaplan JM (1995). Synaptic code for sensory modalities revealed by *C. elegans* GLR-1 glutamate receptor. Nature.

[24] Maricq AV, Peckol EL, Driscoll M, Bargmann CI (1995). Mechanosensory signalling in *C. elegans* mediated by the GLR-1 glutamate receptor. Nature.

[25] Ultsch A (1992). Glutamate receptors of *Drosophila melanogaster*: cloning of a kainate-selective subunit expressed in the central nervous system. Proc. Natl. Acad. Sci. U. S. A..

[26] Kasssimon G, Pannaccione A, Pierobon P (2003). GABA and glutamate receptors are involved in modulating pacemaker activity in *hydra*. Comp. Biochem. Physiol. A Mol. Integr. Physiol..

[27] Boczon K, Myjak P, Wedrychowicz H (2005). Advances in biochemistry and molecular biology of human and animal parasites. Wiad Parazytol..

[28] Li M (2016). The inhibitory role of γ-aminobutyric acid (GABA) on immunomodulation of Pacific oyster *Crassostrea gigas*. Fish Shellfish Immunol..

[29] Zhang G, Li L, Meng J, Qi H, Zhang L (2015). Molecular basis for adaptation of oysters to stressful marine intertidal environments. Ann. Rev. Anim. Biosci..

[30] Wang L, Song X, Song L (2018). The oyster immunity. Dev. Comp. Immunol..

[31] Zhang T (2014). The specifically enhanced cellular immune responses in Pacific oyster (*Crassostrea gigas*) against secondary challenge with *Vibrio splendidus*. Dev. Comp. Immunol..

[32] Lee JS (2016). Glutaminase 1 inhibition reduces thymidine synthesis in NSCLC. Biochem. Biophys. Res. Commun..

[33] Svoboda N, Kerschbaum HH (2009). l-Glutamine-induced apoptosis in microglia is mediated by mitochondrial dysfunction. Eur. J. Neurosci..

[34] Hernandez-Davies JE (2015). Vemurafenib resistance reprograms melanoma cells towards glutamine dependence. J. Transl. Med..

[35] Li M (2016). A glutamic acid decarboxylase (*Cg*GAD) highly expressed in hemocytes of Pacific oyster *Crassostrea gigas*. Dev. Comp. Immunol..

[36] Xin L (2016). The systematic regulation of oyster *Cg*IL17-1 and *Cg*IL17-5 in response to air exposure. Dev. Comp. Immunol..

[37] Sun Y (2014). The immunomodulation of a novel tumor necrosis factor (*Cg*TNF-1) in oyster *Crassostrea gigas*. Dev. Comp. Immunol..

[38] Gao Q (2007). cDNA cloning and mRNA expression of heat shock protein 90 gene in the haemocytes of Zhikong scallop *Chlamys farreri*. Comp. Biochem. Physiol..

[39] Thompson JDT (1997). The CLUSTALX windows interface: flexible strategies for multiple sequence alignment aided by quality analysis tools. Nucleic Acids Res..

[40] Liu Z (2016). The simple neuroendocrine-immune regulatory network in oyster *Crassostrea gigas* mediates complex functions. Sci. Rep..

[41] Wang L (2020). AP-1 regulates the expression of IL17-4 and IL17-5 in the pacific oyster *Crassostrea gigas*. Fish Shellfish Immunol..

[42] Qu T (2014). Identification and functional characterization of two executioner caspases in *Crassostrea gigas*. PLoS ONE.

[43] Zhang H (2008). A novel C1q-domain-containing protein from Zhikong scallop *Chlamys farreri* with lipopolysaccharide binding activity. Fish Shellfish Immunol..

[44] Shun, Y. Analysis of Relative Gene Expression Using Different Real-Time Quantitative PCR. *Acta Agronomica Sinica* (2007).

[45] Livak KJ, Schmittgen TD (2001). Analysis of relative gene expression data using real-time quantitative PCR and the 2− ΔΔCT method. Methods.

[46] Smith PE (1985). Measurement of protein using bicinchoninic acid. Analytical biochemistry.

[47] Lv Z (2018). Comparative study of three C1q domain containing proteins from pacific oyster *Crassostrea gigas*. Dev. Comp. Immunol..

[48] Jiang Q (2013). A scallop nitric oxide synthase (NOS) with structure similar to neuronal NOS and its involvement in the immune defense. PLoS ONE.

[49] Cao A, Mercado L, Ramos-Martinez JI, Barcia R (2003). Primary cultures of hemocytes from Mytilus galloprovincialis Lmk: expression of IL-2Rα subunit. Aquaculture.

[50] Ji YB, Qu ZY, Zou X (2011). Juglone-induced apoptosis in human gastric cancer SGC-7901 cells via the mitochondrial pathway. Exp. Toxicol. Pathol..

[51] Liu HT (2009). Chitosan oligosaccharides attenuate hydrogen peroxide-induced stress injury in human umbilical vein endothelial cells. Pharmacol. Res..

[52] Jin Y, Jorgensen E, Hartwieg E, Horvitz HR (1999). The *Caenorhabditis elegans* gene unc-25 encodes glutamic acid decarboxylase and is required for synaptic transmission but not synaptic development. J. Neurosci..

[53] Pasquali CC (2017). The origin and evolution of human glutaminases and their atypical C-terminal ankyrin repeats. J. Biol. Chem..

[54] Liu S (2020). Glutaminase 1 in mandarin fish *Siniperca chuatsi*: Molecular characterization, expression pattern and function involving in virus replication. Aquaculture.

[55] Aoki C, Kaneko T, Starr A, Pickel VM (1991). Identification of mitochondrial and non-mitochondrial glutaminase within select neurons and glia of rat forebrain by electron microscopic immunocytochemistry. J. Neurosci. Res..

[56] Laake JH, Takumi Y, Eidet J, Torgner IA, Ottersen OP (1999). Postembedding immunogold labelling reveals subcellular localization and pathway-specific enrichment of phosphate activated glutaminase in rat cerebellum. Neuroscience.

[57] Olalla L (2002). Nuclear localization of L-type glutaminase in mammalian brain. J. Biol. Chem..

[58] 58Ganor, Y. & Levite, M. in *Nerve-Driven Immunity: Neurotransmitters and Neuropeptides in the Immune System* (ed Levite, M.) 121–161 (Springer Vienna, 2012).

[59] Wang L, Qiu L, Zhou Z, Song L (2013). Research progress on the mollusc immunity in China. Dev. Comp. Immunol..

[60] Li S (2019). Glutamine protects against LPS-induced inflammation via adjusted NODs signaling and enhanced immunoglobulins secretion in *rainbow trout* leukocytes.

[61] Walker CS (2006). Reconstitution of invertebrate glutamate receptor function depends on stargazin-like proteins. Proc. Natl. Acad. Sci. U. S. A..

[62] Pacheco R (2004). Group I metabotropic glutamate receptors mediate a dual role of glutamate in T cell activation. J. Biol. Chem..

[63] Wang L, Song X, Song L (2018). The oyster immunity. Dev. Comp. Immunol..

[64] Xin L (2015). *Cg*IL17-5, an ancient inflammatory cytokine in *Crassostrea gigas* exhibiting the heterogeneity functions compared with vertebrate interleukin17 molecules. Dev. Comp. Immunol..

[65] Kim MG (2015). Regulation of toll-like receptor-mediated Sestrin2 Induction by AP-1, Nrf2, and the Ubiquitin-Proteasome System in Macrophages. Toxicol. Sci..

[66] Rosani U, Varotto L, Gerdol M, Pallavicini A, Venier P (2015). IL-17 signaling components in bivalves: comparative sequence analysis and involvement in the immune responses. Dev. Comp. Immunol..

[67] Zhang Y, Bhavnani BR (2005). Glutamate-induced apoptosis in primary cortical neurons is inhibited by equine estrogens via down-regulation of caspase-3 and prevention of mitochondrial cytochrome c release. BMC Neurosci..

[68] Antipova TA (2010). Effects of selective anxiolytic afobazole on active caspase-3. Bull. Exp. Biol. Med..

[69] Xu J (2016). Caspase-3 serves as an intracellular immune receptor specific for lipopolysaccharide in oyster *Crassostrea gigas*. Dev. Comp. Immunol..

