# Supplementary material for: The glutaminase (CgGLS-1) mediates anti-bacterial immunity by prompting cytokine synthesis and hemocyte apoptosis in Pacific oyster Crassostrea gigas
Source: Sci Rep. 2021 Jan 14;11:1281. doi: 10.1038/s41598-020-80552-2 (PMC7809476; doi:10.1038/s41598-020-80552-2)
Supplement: Supplementary file 1 — Supplementary figures. [file 41598_2020_80552_MOESM1_ESM.pdf]

The glutaminase (*Cg*GLS-1) mediates anti-bacterial immunity by prompting cytokine synthesis and hemocyte apoptosis in Pacific oyster *Crassostrea gigas*

Yage Liang [a,d], Meijia Li [a,d], Zhaoqun Liu [a, c,d,e], Yuanmei Li [a,d], Lingling Wang [a, c,d, e*], Linsheng Song [a,b,c.e*]

[a] Liaoning Key Laboratory of Marine Animal Immunology, Dalian Ocean University, Dalian 116023, China

[b] Southern Marine Science and Engineering Guangdong Laboratory, Zhuhai 519000, China

[c] Functional Laboratory of Marine Fisheries Science and Food Production Processes, Qingdao National Laboratory for Marine Science and Technology, Qingdao 266235, China

[d] Liaoning Key Laboratory of Marine Animal Immunology and Disease Control, Dalian Ocean University, Dalian 116023, China

[e] Dalian Key Laboratory of Aquatic Animal Disease Prevention and Control, Dalian Ocean University, Dalian 116023, China

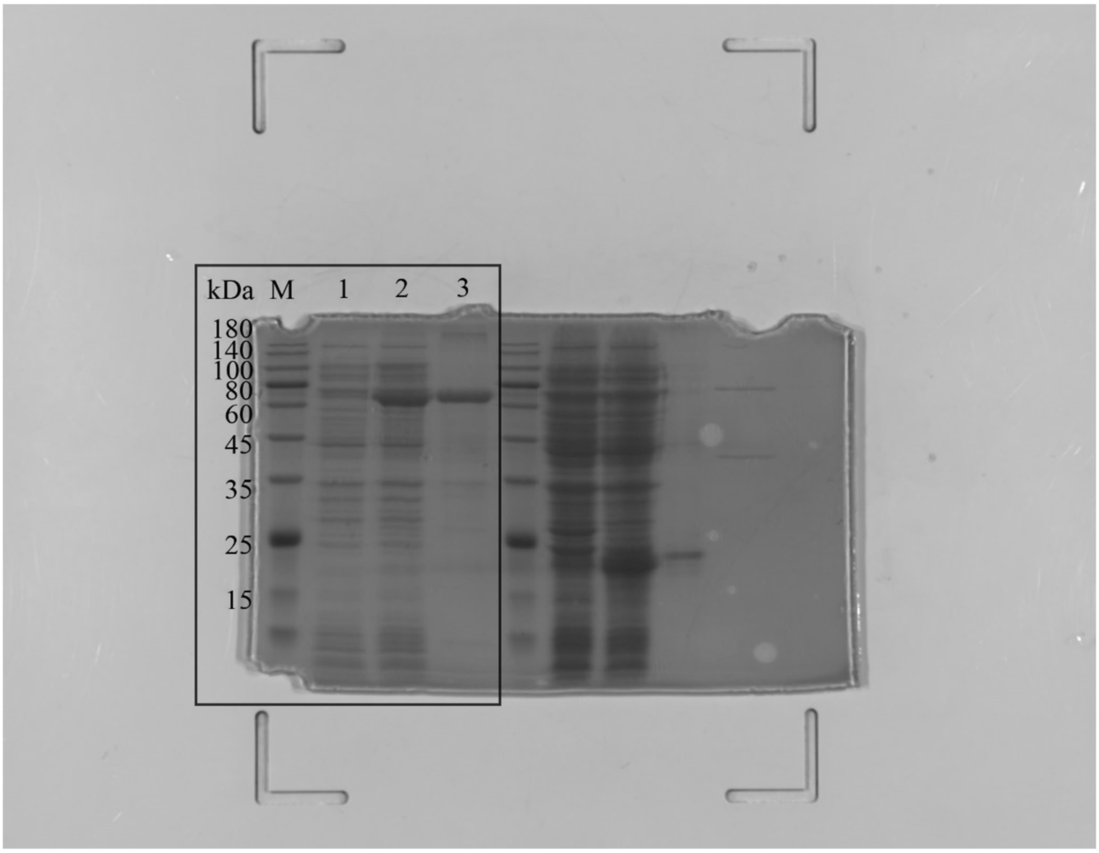

Supplementary Figure 1. SDS-PAGE of analysis rCgGLS-1. Lane M: protein molecular standard (kDa); Lane 1: negative control for r*Cg*GLS-1 (without induction); Lane 2: induced r*Cg*GLS-1; Lane 3: purified r*Cg*GLS-1.

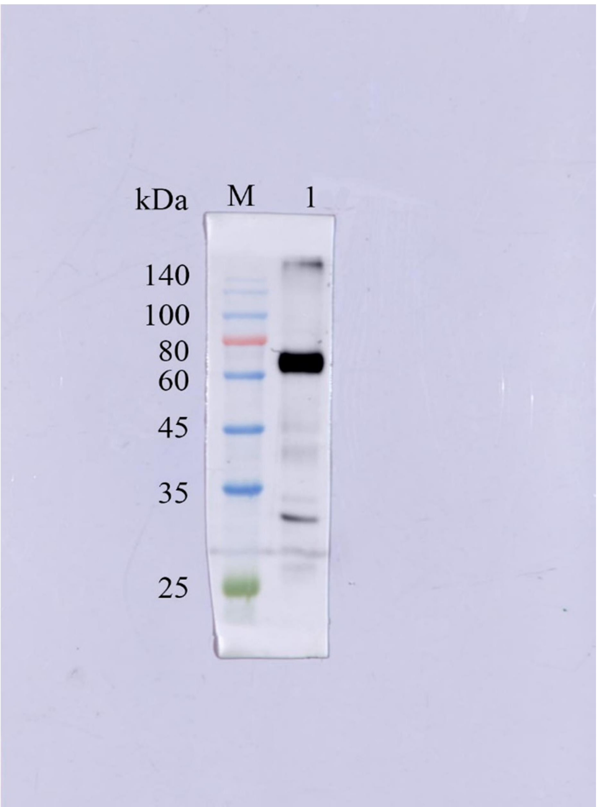

Supplementary Figure 2. Western blot analysis of *anti*-r*Cg*GLS-1. Lane M: protein molecular standard (kDa); Lane 1: Western blot based on the sample of purified r*Cg*GLS-1.
